# High-Dose Insulin Therapy for Refractory Shock in a Polypharmacy Overdose of Calcium Channel Blocker and Oral Hypoglycemic Agent: A Case Report and Literature Review

**DOI:** 10.7759/cureus.77853

**Published:** 2025-01-22

**Authors:** Vinayak S R, Bipenthung N Jami, Austin J Mangaly, Krishnakumar R S, Gladson Vineeth, Chandni Radhakrishnan

**Affiliations:** 1 Emergency Medicine, Government Medical College, Kozhikode, Kozhikode, IND; 2 General Medicine, Government Medical College, Kozhikode, Kozhikode, IND; 3 Internal Medicine, Government Medical College, Kozhikode, Kozhikode, IND

**Keywords:** calcium channel blocker overdose, drug overdose, high-dose insulin therapy, refractory shock, sulfonylurea overdose, toxicology and envenomation

## Abstract

We report a case of polypharmacy overdose involving a calcium (Ca) channel blocker and an oral hypoglycemic agent, where conventional therapies failed to obtain the desired clinical outcome. High-dose insulin (HDI) therapy, a promising treatment regime in this regard, was carried out for our patient, and her condition improved over the subsequent days.

## Introduction

Toxicity due to calcium (Ca) channel blocker overdose, although an uncommon presentation in the emergency department, poses significant challenges to the treating physician [1]. This is because conventional therapies such as IV fluid boluses, vasopressors, and glucagon often fail in severely intoxicated patients [2]. High-dose insulin (HDI) therapy is a promising alternative to such refractory cases with a high risk for mortality [3]. Although high-dose insulin therapy has proven effectiveness, its use in the concomitant intake of an oral hypoglycemic agent has limited evidence. Here, we present to you a case of a 42-year-old woman in refractory shock following amlodipine and glimepiride overdose.

## Case presentation

A 42-year-old woman was brought to our emergency department after allegedly consuming 20 tablets of amlodipine (5 mg) and six tablets of glimepiride (2 mg). She was brought two hours after the incident. On arrival, she was in shock with a blood pressure of 84/60 mm Hg and a heart rate of 100 per minute. A venous blood gas revealed a pH of 7.36 with a bicarbonate value of 18 mmol/L and a partial pressure of carbon dioxide (pCO_2_) of 31 mm Hg. She was initially started on a fluid bolus (0.9% normal saline) of 1 L and later started on a vasopressor (injection noradrenaline) at 4 μg per minute and up-titrated. She was also started on an injection of calcium gluconate (10 mL in 90 mL normal saline) and shifted to the emergency department ICU. Despite these measures, her condition deteriorated as she developed bradycardia (heart rate: 40 per minute) and a further drop in blood pressure (74/50 mm Hg). She was given one dose of IV atropine (1 mg), after which the heart rate improved but hypotension persisted. We started her on high-dose insulin (HDI) therapy, where insulin was administered as an infusion at a dose of 50 U per hour. Dextrose infusion was given concurrently (25% at 80 mL per hour) and titrated according to glucometer random blood sugar (maintained between 100 and 200 mg/dL). Calcium chloride was given at a dose of 20 mL (10%) eight hourly. Over the next 24 hours, her heart rate and blood pressure showed significant improvement, and the dose of insulin was titrated down. Over the next three days, the dose of insulin was gradually titrated down and stopped, alongside noradrenaline and calcium chloride. During the course of treatment, serum electrolytes (sodium {Na}, potassium {K}, Ca, and magnesium {Mg}) were continuously monitored (initially on a four-hourly basis over the first 24 hours), and corrections were given accordingly (Table 1).

**Table 1:** Clinical course in terms of vitals and serum electrolyte levels, since the initiation of high-dose insulin therapy

Day	Time (24-hour format)	Blood glucose (mg/dL)	Heart rate (beats per minute)	Blood pressure (mm Hg)	Serum potassium (mEq)	Serum calcium (mEq)	Regular insulin infusion (U/hour)	25% dextrose (mL/hour)	Noradrenaline (μg/minute)
1	15:30	220	94	80/40	3.6	8.2	50	75	18.7
	17:30	236	93	81/52	3.4	9.9	40	80	17.3
	21:30	180	107	92/57	3.6	12.0	30	90	13.3
2	02:00	250	98	103/60	4.3	11.7	30	80	17.3
	08:00	210	101	107/66	4.4	10.1	30	80	16
	14:00	88	106	99/57	4.5	9.1	15	60	2.7
	22:00	111	93	105/66	3.9	11	8	60	4
3	02:00	237	116	90/55	3.2	10.2	4	100	4.7
	08:00	230	106	106/54	5.3	12.8	4	80	4.7
	14:00	103	90	127/65	4.6	12.2	4	70	4.7
	20:00	252	102	120/65	5.7	13.3	6	50	4.7
4	02:00	159	102	109/66	4.2	13	4	60	4.7
	08:00	122	95	110/71	4.5	13.5	Stopped at 03:00	50	2.7
	14:00	102	107	105/68	4.1	13.3	-	Stopped at 05:00	Stopped at 05:00
	20:00	96	70	101/60	3.9	10.3	-	-	-
5	02:00	86	68	104/60	3.9	10.2	-	-	-
	08:00	157	75	120/76	4.3	11.1	-	-	-

By day 4, she was off all support and was fully conscious and oriented. On day 5, she was shifted out of the ICU and discharged from the hospital on day 8.

## Discussion

The cardiovascular effects of calcium channel blocker overdose are primarily due to their inhibitory action on L-type calcium channels. This causes vasodilation, the inhibition of sinoatrial automaticity, atrioventricular conduction delay, and myocardial depression [1]. In addition, calcium channel blockers in supratherapeutic doses decrease insulin secretion by preventing calcium influx into pancreatic beta cells. This creates a diabetogenic effect of hyperglycemia and metabolic acidosis [4-6].

Although insulin is not a vasopressor, the mechanisms by which high-dose insulin proves effective in calcium channel blocker poisoning include increased inotropy, increased intracellular glucose transport, and vasodilatation [1]. The preferred energy substrate of a stressed myocardium is glucose, rather than fatty acids. Insulin in higher doses promotes intracellular glucose transport, facilitating an increase in inotropy [3]. In addition, it directly affects certain intracellular mechanisms that contribute to increased inotropy, especially those involving calcium handling and the P13K pathway [7,8]. By enhancing endothelial nitric oxide synthase activity and its effect on an insulin intracellular signalling pathway (P13K), vasodilation, primarily on the systemic, pulmonary, and coronary vasculature, occurs. This causes a reduction in vascular resistance caused by microvascular dysfunction (caused by cardiogenic shock) and ultimately results in enhanced cardiac output [9].

Despite the challenge of hypoglycemia and concurrent intake of an oral hypoglycemic agent, the titration of dextrose infusion in accordance with timely general random blood sugar (GRBS) monitoring ensured optimal blood glucose range over the course of treatment. While case trials comparing the efficacy to other treatments are lacking in current literature, multiple case reports and case series demonstrate the efficacy of HDI therapy with minimal clinically significant adverse effects [10-12]. Experimental studies have proven increased survival while using HDI in verapamil poisoning in dogs [13]. In a case report by Greene et al., only one person out of seven developed hypoglycemia, which was immediately rectified and clinically insignificant [14]. Although there was one case report by Corcoran et al., where the patient required prolonged HDI therapy (37 hours) at 10 U/kg/hour, serum insulin levels remained supraphysiologic beyond 24 hours after the termination of therapy [15]. In our case, although the dose of HDI was lower, the duration was longer. Although dextrose infusion rates remained slightly higher when insulin infusion rates were lowered, further dextrose infusion was not required two hours beyond the stoppage of insulin infusion. In our situation, since the patient was refractory to conventional therapy, we had to resort to this alternative treatment, which improved the patient's condition dramatically. With this case, we hope to create a positive impact in the current practice of managing patients with severe calcium channel blocker toxicity in polypharmacy overdose.

## Conclusions

High-dose insulin therapy can be an effective treatment for calcium channel blocker toxicity, even when there is a concomitant intake of an oral hypoglycemic agent. This case report highlights the potential benefits of this therapy and supports its consideration in patients who are refractory to conventional treatment. Further research is needed to compare the efficacy of high-dose insulin therapy to other treatments for calcium channel blocker toxicity.
